# Synthesis of Thiol-Modified Hemicellulose, Its Biocompatibility, Studies, and Appraisal as a Sustained Release Carrier of Ticagrelor

**DOI:** 10.3389/fphar.2021.550020

**Published:** 2021-05-19

**Authors:** Muhammad Zaman, Rabia Imtiaz Bajwa, Omer Salman Qureshi, Atta Ur Rehman, Sadaf Saeed, Muhammad Wahab Amjad, Maria Abdul Ghafoor Raja, Muhammad Ajaz Hussain

**Affiliations:** ^1^ Faculty of Pharmacy, University of Central Punjab, Lahore, Pakistan; ^2^ Department of Pharmaceutics, Faculty of Pharmacy, The University of Lahore, Lahore, Pakistan; ^3^ Department of Pharmacy, Forman Christian College, Lahore, Pakistan; ^4^ Faculty of Pharmacy, Northern Border University, Arar, Saudi Arabia; ^5^ Department of Chemistry, University of Sargodha, Sargodha, Pakistan

**Keywords:** hemicellulose, thiourea, thiolation, mucoadhesion, drug release, in vivo analysis

## Abstract

**Background:** Nature has always been considered as the primary source of pharmaceutical ingredients. A variety of hemicelluloses, as well as their modified forms, have been under investigation. Herein, a study was designed to explore the biocompatibility of hemicellulose and its modified form (thiolated hemicellulose) as well as its potential as a pharmaceutical excipient.

**Method:** For thiol modification thiourea was used as the thiol donor, HCl as the catalytic reagent, and methanol was used for washing purposes. Modified polymers were characterized for physicochemical characteristics, including surface morphology, the amorphous or crystalline nature of the particles, modification of polymer by FTIR, and biocompatibilities. For acute oral toxicity study, a single dose of 2 g/kg was administered to albino rats of 200 g average weight (*n* = 3). Polymers were evaluated as pharmaceutical excipients by preparing compressed tablets of antiplatelet drug (Ticagrelor), followed by various quality control tests, such as swelling index, thickness and diameter, disintegration, and *in-vitro* drug release.

**Results:** From the results, it was observed that thiol modification has been successfully accomplished as characteristic peaks belonging to –SH group appeared at 2667.7691 cm^−1^ in FTIR scan. The modified polymer was found safe in the use concentration range, confirming their safe use for *in vivo* analysis. No significant effect has been observed in the behavior, biological fluid (blood), or on vital organs. Thiolated hemicellulose was found to be an excellent drug retarding polymer as 8 h of dissolution studies showed that 67.08% of the drug has been released.

**Conclusion:** Conclusively, incorporation of thiol moiety made the polymer more mucoadhesive with, and a worthy carrier of, the drug with good biocompatibilities.

## Introduction

Research into the field of drug discovery and drug development by professional teams of pharmacists, scientists, clinicians, and statisticians is being done to contribute toward the betterment of human care. Such advance research offers great potential for improving diagnosis, treatment, and mitigation of diseases. Today’s pharmaceutical industry offers many reliable formulation strategies by altering polymers for Biopharmaceutical Classification System class IV drugs, which have reduced the roadblock toward development of dosage forms. Any modification done in the polymer’s structure will upgrade its features, thus providing desirable outcomes (Pillai and Panchagnula, 2001). Research into new drug deliveries has widely used polymers as an integral part in the development and advancement of medicine. They are being fabricated in hydrogels, microspheres, nanoparticles, and modified release tablets, providing controlled and targeted drug delivery, thus enhancing the efficacy. Many nano-sized drug deliveries like liposomes, dendrimers, and polymeric micelles have also been developed, displaying attractive properties (Kadajji and Betageri, 2011). Present research involves modification done in polymeric structures for the purpose of providing vast applications in controlled drug release. Controlled and targeted drug deliveries using polymers offer the most potential opportunities, hence giving a better cyclic dosage (Liechty et al., 2010). Nowadays, many efforts are being made to increase the absorption of less soluble and less permeable drugs through mucosal membranes. Some of these efforts involve the conjugation of a drug with a thiomer, which greatly improves the mucoadhesive properties following enhanced bioavailability. This modified generation of polymers is considered to increase the mucoadhesive properties by 2–140 folds (Hanif et al., 2015). The mucoadhesive drug delivery system plays an important role in the controlled release of various therapeutic agents. Since polymers contain several functional groups, these functional groups combine to form amide and ester linkages with mucin molecules of mucosae to get better mucoadhesion of thiomers (Bernkop et al., 1999). There are a large number of mucoadhesive polymers that are being widely used like chitosan, arabinoxylan, sodium alginate, carbopol, eudragit analogues, pectin, gelatin, and acacia. Out of these some are considered as novel mucoadhesives e.g. lectins, poloxamer, alginate-polyethylene glycol acrylate, and thiolated polymers including chitosan–iminothiolane, poly (acrylic acid)–cysteine, poly (acrylic acid)–homocysteine, chitosan–thioglycolic acid, alginate–cysteine, and poly (methacrylic acid)–cysteine (Mythri et al., 2011). Many chemical modifications have been done in natural polysaccharides to achieve significant returns. Thiol modification of natural polysaccharides such as chitosan, alginate, pectin, and tamarind seed xyloglucan has been successfully done to improve their functional properties. In pharmaceutical applications these modified polymers are effectively working as drug carriers (Bhatia and Ahuja, 2013). Ispaghula seed husks carry a large number of xylans (arabinoxylans) that are actually hemicelluloses and are found both in primary and secondary cell walls of plants. They are easily extracted via a simple extraction process like alkali extraction or hot water extraction (Saghir et al., 2008). Hemicellulose is mainly used as a mucoadhesive polymer and the strength of mucoadhesion is enhanced by modifying the structure via attaching a thiol moiety in the polymeric backbone (Zaman et al., 2018a).

The current study aimed to achieve extraction of hemicellulose from Ispaghula husk, successful thiolation of hemicellulose, evaluation of physicochemical properties, and acute toxicity studies of thiolated hemicellulose in comparison to natural polymer. This was followed by the development of controlled release compressed tablets of Ticagrelor to overcome its bioavailability problems, as its belongs to the Biopharmaceutical Classification System (BCS) class IV drugs and has a lower oral bioavailability (approximately 36%) (Dobesh and Oestreich, 2014).

## Materials and Methods

### Materials

Ispaghula husk was purchased from the local market of Lahore, Pakistan. Thiourea, ethanol, Methanol, potassium chloride, and hydrochloric acid, all from Merck, Darmstadt Germany, were obtained from the research laboratories of The University of Lahore. Ellman’s Reagent (5,5-dithio-bis-(2-nitrobenzoic acid) was purchased from Sigma-Aldrich Gmbh Chemie, Germany. Distilled water was obtained from the research laboratory of The University of Lahore. All the chemicals and reagents used were of analytical grade.

### Methods

#### Alkali Extraction of Hemicellulose

**FIGURE 1:**
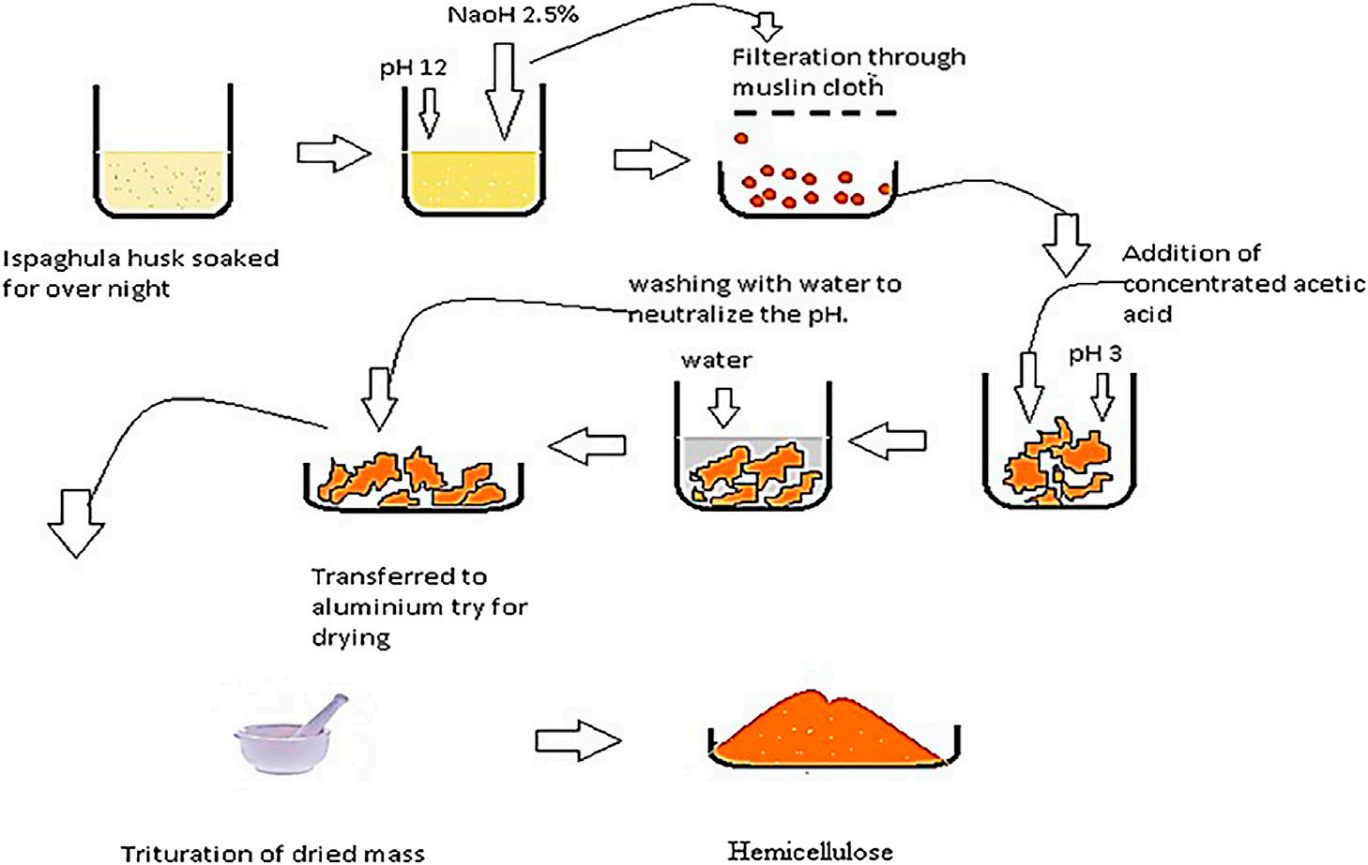
Pictorial description of alkali extraction, used for the extraction of Hemicellulose from Ispaghula husk.

Extraction of hemicellulose was done from *Plantago Ovata* using alkali extraction technique. 100 g of *Plantago ovata*, commonly known as Ispaghula Husk, was taken in a beaker and soaked in distilled water. 2.5% aqueous solution of NaOH was used to adjust the pH to 12. The gel formed was filtered twice from the mixture using a Muslin cloth to separate out husk as a residue; pH was decreased to three by adding concentrated acetic acid, which resulted in coagulation of the gel (Figure 1). Multiple washing of the coagulated gel was done with distilled water to neutralize the pH. Finally, the gel was dried in an oven at a temperature of 40°C to get the dried product (Zaman, 2018).

#### Percent Yield of Hemicellulose

The percent yield of Hemicellulose was calculated as follows:

$$\%\;\text{yield}=\frac{\text{x}}{\text{y}}\times 100,$$ (1)

where x = Weight of hemicellulose obtained and y = Weight of Ispaghula husk.

#### Thiolation of Hemicellulose

**FIGURE 2:**
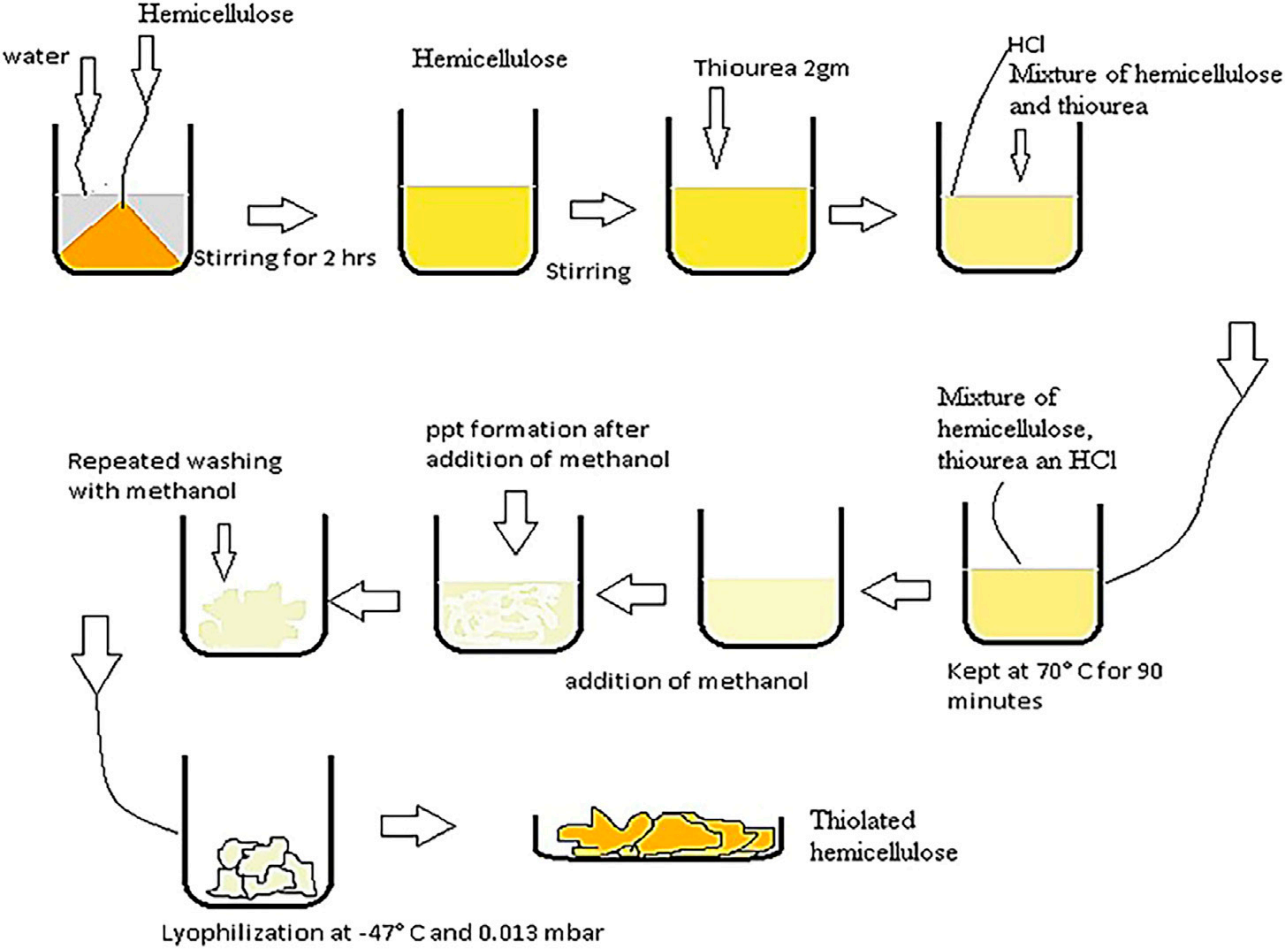
Schematic diagram, describing the process for thiolation of hemicellulose.

An ester linkage was developed between polymer and the thiol group, donated by thiourea. The reaction was carried out in the presence of hydrochloric acid (HCl), that served as a catalyst to mediate the chemical reaction. In an 100 ml beaker containing 50 ml of distilled water, 1 g of hemicellulose was added and stirred for 2 h at room temperature. After hydrating the polymer, 2 g of thiourea was added to the hydrated polymer and stirred for 10 min. This was followed by addition of catalytic amounts (4–5 drops) of HCl to carry out the reaction. This reaction mixture was stirred for another 5 min on a magnetic stirrer to get a homogenized product. Later on, the mixture was kept at 70°C for 90 min. After the 90 min, methanol was added to the mixture to cool it down, till the formation of white precipitates. These precipitates were washed multiple times with methanol to remove any unreacted thiourea (Figure 2). The resultant mixture was then cooled at −80°C, followed by lyophilization for 48 h at −47°C and 0.013 mbar pressure to get the dried product (Bhatia and Ahuja, 2013).

#### Physicochemical Properties of Hemicellulose and Thiolated Hemicellulose

##### Water Solubility

Hemicellulose isolated from Ispaghula husk and thiolated hemicellulose was checked for its solubility in water. For this purpose, 0.5 g of hemicellulose was weighed and transferred to a beaker containing 10 ml of 95% ethanol for water solubility purpose. The mixture was stirred for 2 min. Then, 90 ml of water was added with continuous stirring until a solution was formed. This solution was kept at room temperature for 2 days, which formed a gel (Saghir et al., 2008). Whereas 100 mg of thiolated hemicellulose was allowed to dissolve in 50 ml distill water under overnight stirring to check its aqueous solubility.

##### pH of Aqueous Solution/Dispersions

One (1) % (w/v) aqueous dispersions of thiolated and non-thiolated polymer were prepared in distilled water. pH meter’s probe was dipped in the solution till the constant value and pH was noted.

##### Loss on Drying

One gram of hemicellulose and thiolated hemicellulose was taken and loaded separately in a digital moisture analyzer that was run under specified conditions and LOD was noted.

##### Swelling Index

One gram of hemicellulose and thiolated hemicellulose were taken separately in graduated cylinders with capacities of 50 cm^3.^ Initial volumes were noted (V_i_). Then, distilled water was used to make up the volume 50 ml in both cylinders. Maximum swelling had been observed when the mixture was allowed to stand overnight. Final volume (V_f_) was noted. Swelling index of both polymers was calculated using the following equation.

$$\text{SI}=\frac{{\text{V}}_{\text{f}}-{\text{V}}_{\text{i}}}{{\text{V}}_{\text{i}}}\times 100.$$ (2)

Here, V_f_ is the final volume and V_i_ is the initial volume.

##### Micromeritic Studies

Both, the modified and un-modified polymers were evaluated for their flow properties. A comparative analysis was done for bulk densities, tapped densities, compressibility indexes, Hausner’s ratios and the angle of repose.

##### Determination of Thiol Content by Ellman’s Reagent Method

The thiol contents were determined by Ellman’s reagent technique. One (1) ml of 0.2% w/v solution of thiourea was prepared by dissolving it in 0.5 M PBS of pH 8, followed by preparation of serial dilution in the concentration range of 10 to 100 μg/ml. The prepared dilutions were analysed spectrophotometrically at 280 nm.

The thiol group, attached with the polymeric back bone was determined by Ellman’s Reagent technique, and for said purpose a linearity graph of thiourea has been constructed. Initially, a 0.2% standard stock solution of thiourea prepared using PBS as solvent, followed by the preparation of serial dilutions ranging from 10 to 100 μg/ml. Later on, these were subjected to spectrophotometric analysis at 280 nm

Thiolated hemicellulose was evaluated for thiol content spectrophotometrically by applying the Ellman’s reagent method (Hornof et al., 2003). The thiolated polymer was dissolved in the sufficient quantity of the demineralized water obtained 2% w/v solution. On the other hand, 0.5 M PBS of pH 8 was used to prepare 150 µl of Ellman’s reagent. For this, 3.96 mg of the reagents were taken and 10 ml solution was prepared in PBS. In a separate beaker, 150 µl of Ellman’s reagent in 1350 µl of PBS and 150 µl of thiolated polymer in 1350 µl of deionized water were mixed, and incubated at room temperature for 10 min. Afterward the sample was taken and absorbance was measured spectrophotometrically at 412 nm (Zahir-Jouzdani et al., 2018). Thiourea calibration curve was used to calculate free thiol groups attached to the polymer.

##### Fourier Transformed Infrared Spectroscopy (FT-IR)

Attenuated Total Reflectance Fourier Transformed Infrared Spectroscopy (ATR-FTIR) was performed on FTIR spectrophotometer 7,600 to ensure the compatibility of ingredients. These studies were carried out on pure hemicellulose and prepared polymer (Thiolated hemicellulose). The scanning was done at a range of 400–4000 cm^−1^ using ATR-FTIR technique (Parmar et al., 2010).

##### Surface Morphology Studies

The surface morphology of thiolated as well as non-thiolated hemicellulose was observed by scanning electron microscope (SEM JSM5910 JEOL, Japan) at different magnification powers (500X, 1000X and 2500X). The small quantity of the polymeric material has been placed on the stage of SEM and images were taken (Rao et al., 2017).

##### X-Ray Diffractometry

In order to observe the effect of thiolation on the crystalline or amorphous structure and relative configuration of the polymer, an x-ray diffractometric analysis has been performed using X-ray diffractometer (JDX-3532 JEOL, Japan). The environment of the instrument was maintained as, voltage of the tube at 45 kV with 40 mA tube current and scanning angle 2θ was 5–50° (Yu et al., 2006).

##### Acute Toxicity Studies of Polymers

The acute toxicity studies for hemicellulose and its modified form were performed according to the guidelines provided by the Organization for Economic Co-operation and Development (OECD), using Albino rats (Jaykaran et al., 2009). The animal experiment was approved by the Institutional Research Ethics Committee of The University of Lahore under the project file no. IREC-2019-101.

###### Animal

Fifteen (15) albino rats having individual average weight of 200 g were purchased from the animal house, Faculty of Pharmacy, The University of Lahore. The animals were placed in clean cages and kept in 12 h day/night cycle under controlled laboratory conditions. The animals were provided with a conventional lab diet and water ad libitum.

###### Preparation of Test Animal

The animals were kept in the clean cages and placed in the lab for a week to get climatized with the environment. After that, the animals were randomly divided in three groups: Group 1 (control), Group 2 (hemicellulose treated) and Group 3 (thiolated hemicellulose treated).

###### Acute Oral Toxicity

• Preparation of Test Substances to be Administered for Oral Toxicity

According to OECD guidelines of acute toxicity, test substances to be administered were moistened according to the weight of each rat. The volume for rodents should not exceed 1 ml/100 g of body weight, thus the volume used was chosen according to the weight of each rat for the purpose of noting any kind of GIT disturbances (Additives E.P.o.F. and Food N.S.a.t., 2013). A single dose study was conducted by administering 50, 300, and 2000 mg/kg of body weight of the animal (Lodhi et al., 2020). The groups of the animals were administered with the both hemicellulose and thiolated hemicellulose, accordingly, and monitored carefully for any kind of illness or if any of the animals died during the experiment. The rats from each group were administered with the required volume, depending upon the body weight, via feeding tube. Later on, continuous observations were made for the first 4 h and then on the third, seventh, and 14th day. The number of rats that survived the study was noted and a necropsy was done on each rat at the 15th day.

###### Body Weight

The body weight of the individual animals of all three groups were determined before dosing, after 1st, 7th and 14th day after dosing. The gain or loss in weight of treated groups were compared to that of control group.

###### Mortality

The animals of all groups kept under close observation on daily basis to notice the any sort of abnormal changes such as diarrhoea, tremors or behaviour.

###### Hematological and Biochemical Analysis

Blood sample from individual rat was drawn and processed for haematological as well biochemical analysis. The outcomes were reported and interpreted.

###### Necropsy

The survived rats were anesthetized by Intraperitoneal injection and processed for complete necropsy. The vital organs, such as kidney, liver and heart were observed inflammation or lesions.

###### Relative Organ Weight

The relative weight of the liver, kidney and heart was determined by following mathematical expression:

$$\text{Relative Organ Weight}\left(\text{ROW}\right)=\frac{\text{Absolute organ weight }\left(\text{g}\right)}{\text{Body weight of rat on the day of sacrifice }\left(\text{g}\right)}\times 100$$ (3)

Later on, the organs were preserved in 10% Formalin solution for further histopathological analysis (Erum et al., 2015).

#### Preparation of Ticagrelor Sustained Release Tablets

A commonly used direct compression method was employed for the preparation of sustained release tablets. Two formulations, F1 and F2 were prepared using hemicellulose and thiolated hemicellulose as sustained release polymers, respectively. All the other excipients including lubricant (talc), glidant (magnesium stearate), binder (polyvinylpyrrolidone), flow modulator (avicel pH 102) and sweetener (aspartame) were weighed carefully using digital weighing balance and pass through sieve # 40 (Table 1). All the ingredients, excluding talc and magnesium stearate, were mixed for 15 min using pestle and mortar, followed by addition of lubricant and glidant, and mixing for additional 5–10 min. Finally, the blend was subjected to direct compression using single punch tablets compression machine.

**TABLE 1:** Composition of modified release tablets of Ticagrelor.

Ingredients	F1 (mg)	F2 (mg)
Ticagrelor	90	90
Polymer	75	75
PVP k-30	12.5	12.5
Mg-stearate	2.5	2.5
Talc	2.5	2.5
Aspartame	7.5	7.5
Avicel pH 102	57.3	57.3

##### Post Compression Studies for Ticagrelor Sustained Release Tablets

Following post compression, tests were performed on Ticagrelor modified release tablets.

##### Diameter and Thickness

Ten (10) tablets from each formulations were selected randomly for the determination of thickness and diameter using digital vernier caliper. The outcomes were recorded as mean and standard deviation (Afifi and Ahmadeen, 2012).

##### Weight Variation Test

Twenty (20) tablets were randomly selected from both formulations and the weight variation test was performed using digital weighing balance. The results were compared with the official limits provided in US Pharmacopoeia (Bayoumi, 2018). The weight variation was calculated in percentage by:

$$\text{W}\text{.V}=\frac{{\text{I}}_{\text{w}}-{\text{A}}_{\text{w}}}{{\text{A}}_{\text{w}}}\times 100,$$ (4)

where W.V is the weight variation and I_w_ and A_w_ were individual and average weight respectively.

##### Hardness

The Monsanto Hardness Tester was used to determine the hardness of randomly selected 6 tablets from each formulation. The hardness provide the indication of mechanical resistance against breakage and erosion during packing, transportation and storage (Jain et al., 2019).

##### Friability

Ten tablets were selected randomly from each formulation, weighed and transferred to the Roche Friabilator. The apparatus was run for 4 min for 100 revolution at 25 RPM. The tablets were taken out from the friabilator, de-dusted and their weight was determined again. The percentage friability was calculated as (Chavan et al., 2018):

$$\text{F}=\frac{{\text{I}}_{\text{w}}-{\text{F}}_{\text{w}}}{{\text{I}}_{\text{w}}}\times 100,$$ (5)

where I_w_ = initial weight and F_w_ = final weight.

##### Disintegration Test

Six (6) tablets were randomly selected from each formulation and subjected to disintegration test using disintegration apparatus having a basket rack assembly. The temperature of the apparatus was set to 35–39 °C. After the completion of specified time duration, the basket was lifted and observed for disintegration. The time for complete disintegration of the tablets was determined (Kambham and Bonnoth, 2016).

##### Swelling Index

A tablet from each formulation was selected, weighed and subjected to determine the swelling index. It provide information about the extent to which a tablet can absorb and retain the moisture. The tablet was positioned on a tarred glass slide and placed in a petri dish having 10 ml of acidic medium of pH 1.2. After different time intervals (15 min, 30 min, 45 min, 1 h, 2 h, 4 h, 6 h, and 8 h, 24 h) till the constant weight achieved. The swelling index was determined using the following equation (Patel et al., 2007):

$$\text{S}\text{.I}=\frac{{\text{W}}_{2}-{\text{W}}_{1}}{{\text{W}}_{1}}\times 100,$$ (6)

where S.I was swelling index and W_2_ and W_1_ were final and initial weights respectively.

##### Percent Drug Contents

###### Preparation of Standard Solution

90 mg of Ticagrelor was accurately weighed and dissolved in hydrochloric acid buffer solution of pH 1.2 in 100 ml volumetric flask.

###### Preparation of Sample Solution

10 tablets from each formulation were taken and crushed. An amount of powder equivalent to 90 mg drug was taken and dissolved in 100 ml volumetric flask containing hydrochloric acid buffer solution of pH 1.2.

Both the solutions were analyzed spectrophotometrically at a wavelength of 222 nm. Dilution factor was applied and drug content was calculated by the following mentioned formula (Lastra et al., 2003):

$$\%\text{Drug}\;\text{Content}=\frac{\text{Absorbance}\;\text{of}\;\text{sample}}{\text{Absorbance}\;\text{of}\;\text{standard}}\times \frac{\text{Concentration}\;\text{of}\;\text{standard}}{\text{Concentration}\;\text{of}\;\text{sample }}\times 100.$$ (7)

##### *In-Vitro* Drug Release Studies

To study the release of drug from prepared tablets of Ticagrelor, dissolution was carried out in USP Type II Dissolution paddle apparatus. 900 ml of 0.1 N hydrochloric acid buffer of pH 1.2 was used as the dissolution media. Tablets were transferred and apparatus was run at 50 RPM, 37°C ± 0.5°C. 5 ml of sample was withdrawn periodically at 2 min, 5 min, 10 min, 15 min, 20 min, 30 min, 1 h, 2 h, 4 h, 6 h, and 8 h with the replacement of an equal volume of fresh dissolution medium and was diluted for further analysis. The prepared samples were analyzed spectrophotometrically at 222 nm (Hossain et al., 2013).

##### Kinetic Modeling

Kinetic modeling was performed to check the mechanism and behavior of the released drug from sustained release tablets. Different kinetic models, namely Zero order, first order, Higuchi, and Korsmeyer Peppas models, were applied to find out the release behavior of the drug.

##### Mucoadhesion Strength

Mucoadhesion strength of formulations F1 (Hemicellulose) and F2 (Thiolated hemicellulose) based tablets were performed using Texture analyzer. Apparatus consisted of 50 N load cell equipped with a mucoadhesive holder. Formulated tablets were allowed to attach to a cylindrical probe of 10 mm diameter with the aid of double sided adhesive tape. Freshly excised mucosa of rabbit was taken and cut into pieces. Before placing the membrane pieces on the mucoadhesive stage holder, the mucosa was equilibrated for 15 min at 37.0 ± 0.5°C. A contact was made between the tablet attached to a probe and the mucosa membrane by lowering the probe at a rate of 0.5 mm/s, followed by maintaining a contact force of 1 N for 60 s. After 60 s the probe was subsequently withdrawn at the same rate at a distance of 15 mm. Thus, the maximum force required to separate the probe from mucosa membrane was calculated using Texture Exponent 32 Software.

#### Statistical Analysis

Statistical analysis was performed using graph pad prism version 7.01. The analysis of variances (ANOVA) with 95% of confidence interval was applied, followed by Tukey’s multiple compression test. The test was performed on release data to observe the difference in sustained effect of both thiolated and non-thiolated polymers.

## Results and Discussions

### Percent Yield of Hemicellulose

The production of hemicellulose was via an alkali extraction process carried out on Ispaghula husk. The percentage yield of hemicellulose isolated from the total weight of Ispaghula husk was calculated to be 37 ± 1.2%. The yield obtained in this research was less than the yield obtained and reported earlier in previous literature. There could be several possible reasons for getting a lower yield, one of which could be the use of muslin cloth for filtration purposes instead of using vacuum filtration done through a sintered glass crucible (Saghir et al., 2008).

### Thiolation of Hemicellulose

Polymers were modified to enhance their characteristics. A thiol group via thiourea was attached to the polymer backbone of Hemicellulose. An ester linkage was formed between the polymer Hemicellulose and Thiourea. Successful thiolation was confirmed by FTIR studies and SEM studies, and thiol content was determined by the Ellman’s reagent method by spectrophotometer analysis (UV-1800 Shimadzu). Dialysis method, as explained by Zaman et al. (2018b), was used to drain out the thiourea as it is water soluble and the sample’s water-washing was verified by a negative wet laboratory test (lead sulfide test) of thiourea.

### Physicochemical Properties of Hemicellulose and Thiolated Hemicellulose

Physicochemical properties of modified and un-modified polymer were observed and a comparison was made between the thiolated and non-thiolated polymer (Table 2).

**TABLE 2:** Physicochemical properties of Hemicellulose and Thiolated hemicellulose.

Parameters	Hemicellulose ± S. D	Thiolated hemicellulose ± S. D
Water solubility	Insoluble	Soluble
pH (1% solution/Gel)	7.65 ± 0.01	7.36 ± 0.01
Loss on drying (%)	11.1 ± 0.852	8.5 ± 0.32
Swelling index (%)	55.76 ± 0.73	84.4 ± 2.08

#### Water Solubility

Hemicellulose is a natural swellable polymer. It has an appreciable water holding capacity and is insoluble in water; however, 100 mg of thiolated hemicellulose when allowed to dissolve in 50 ml water showed aqueous solubility.

#### pH of Aqueous Solution/Dispersions

Aqueous dispersions of 1% w/v of both polymers (thiolated and non-thiolated) were prepared and their pH was checked. Hemicellulose showed pH at 7.65 ± 0.01, whereas thiolated hemicellulose showed pH at 7.36 ± 0.01. This slight change in pH could be due to the presence of thiourea residues.

#### Loss on Drying

Previous literature had reported about 10% loss on drying of hemicellulose. The current study investigated that hemicellulose showed 11.1 ± 0.852% loss on drying while thiolated hemicellulose displayed 8.5 ± 0.32% loss on drying. The results obtained were comparable to the studies conducted by Iqbal et al. (2011).

#### Swelling Index

Hemicellulose is a water swellable polymer. Swelling properties of hemicellulose were analyzed and revealed its characteristic water holding capacity. The swelling index for hemicellulose was found to be 55.76 ± 0.73%. Thiolation done on hemicellulose evinced an increase in the swelling property of polymer. It showed an increased swelling index of 84.41 ± 2.08%. SH groups’ incorporation alters the hydrogen bonding systems and creates more ion repulsion and more fluid than comes in the matrix; this might be the reason for an increased swelling of the polymer (Farid-ul-Haq et al., 2020).

Thus, the modification done to the hemicellulose powder via thiol attachment in the polymeric backbone enhanced the swelling strength of the hemicellulose, which was also reported in earlier studies done by Prüfert et al. (2017).

### Micromeritic Studies

Micromeritic studies were carried out to study the flow properties of both thiolated and non-thiolated polymers which were later compared to check the effects of thiolation that appeared on the polymer. The bulk density and tapped density observed for hemicellulose was found to be 0.555 ± 0.001 cm^3^/mL and 0.740 ± 0.003 cm^3^/ml, respectively. The Hausner’s ratio and Carr’s index were calculated to be 1.34 ± 0.030 and 33.33 ± 1.2, respectively. Values obtained from Hausner’s ratio, Carr’s index, and Angle of Repose that happened to be 34.99 ± 0.145 revealed poor flow properties of hemicellulose. In contrast to hemicellulose, thiolated hemicellulose expressed bulk and tapped density as 0.625 ± 0.003 and 0.714 ± 0.001 respectively. Hausner’s ratio and Carr’s index were found to be 1.14 ± 0.010 and 14.28 ± 0.141. Angle of Repose was calculated to be 29.65 ± 0.210. This indicates good flow properties of thiolated hemicellulose via values acquired from Hausner’s ratio, Carr’s index, and Angle of Repose (Table 3). This might be due to the fact that modified polymer has less moisture content, exhibiting a more dried nature. The comparatively greater dried nature of the thiolated particle might allow each other to move a bit briskly.

**TABLE 3:** Micromeritics analysis of hemicellulose and thiolated hemicellulose.

Parameters	Hemicellulose ± S. D (*n* = 5)	Thiolated hemicellulose ± S. D (*n* = 5)
Bulk density (g/cm^3^)	0.55 ± 0.001	0.625 ± 0.003
Tapped density (g/cm^3^)	0.74 ± 0.001	0.714 ± 0.001
Hausner’s ratio	1.34 ± 0.030	1.14 ± 0.010
Carr’s index (%)	33.33 ± 1.21	14.28 ± 0.141
Angle of repose (°)	34.99 ± 0.145	29.65 ± 0.210

#### Determination of Thiol Contents by Ellman’s Reagent Method

**FIGURE 3:**
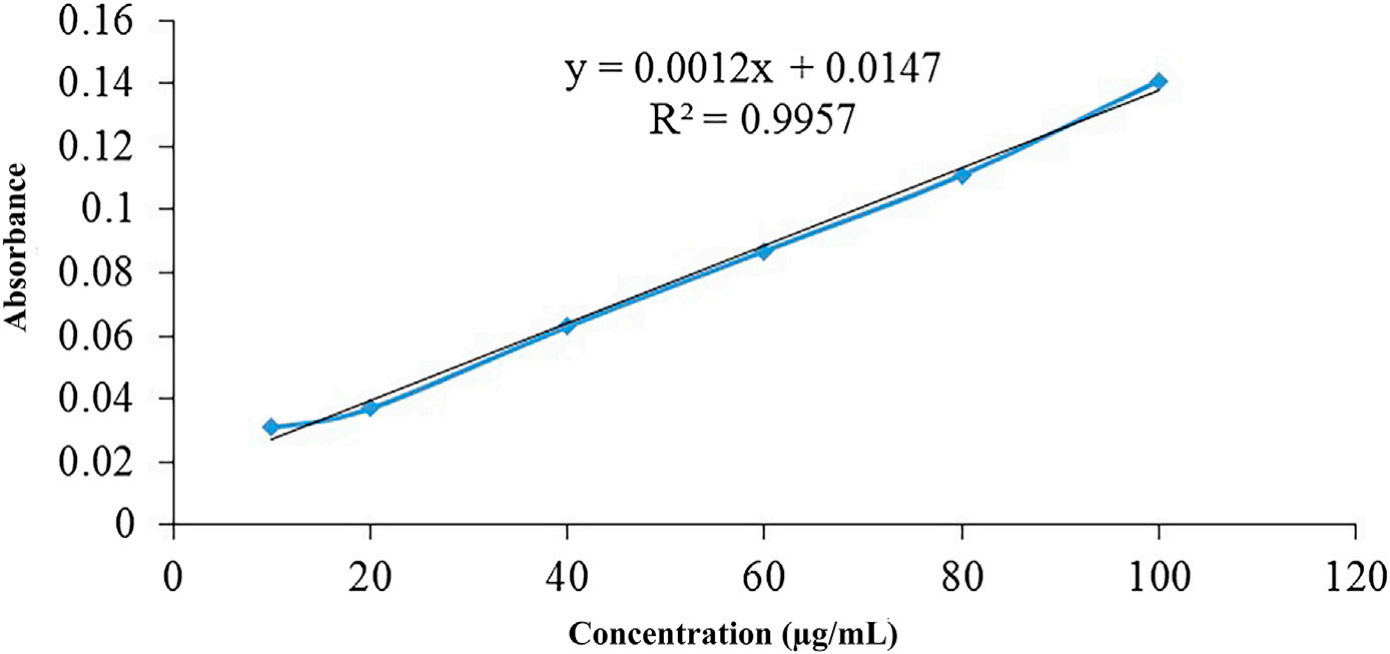
The calibration curve of thiourea was obtained by using serial dilutions, ranging from 10 to 100 µg, and analyzing them by a spectrophotometer at 280 nm wavelength. R^2^ was found to be 0.9957, which demonstrates a good linearity.

Ellmans reagent is hydrophilic in nature and easily reacts with free thiol to produce a yellow color product because it has the ability to react with sulfhydryl group -SH at neutral pH. Sulfhydryl group can be estimated by using a calibration curve of sulfhydryl groups containing compound (Figure 3). Thiol content for thiolated hemicellulose was found to be in the range of 38.375 mmoles per Gram of the polymer and 21.625 mmoles per Gram of the polymer using calibration curve of thiourea.

#### Fourier Transformed Infrared Spectroscopy

**FIGURE 4:**
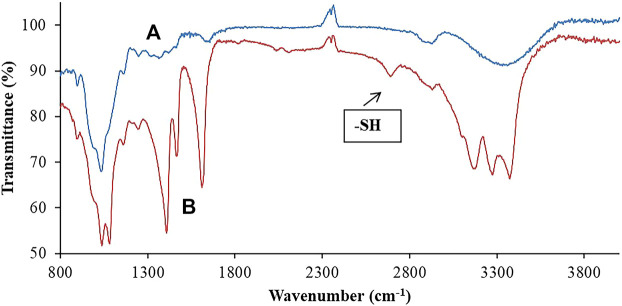
Comparative illustration through FTIR scans of both **(A)** hemicellulose and **(B)** thiolated hemicellulose, confirming the occurrence of chemical changes in the structure of hemicellulose.

FTIR analysis was performed for modified polymers in comparison to unmodified polymers to confirm the success of thiolation. FTIR spectrophotometer 7,600 helped in observing the specific bonds and chemical structure of thiolated and non-thiolated polymers. A broad absorption band was observed in hemicellulose at 3370.9603 cm^−1^ conferring to –OH stretch of alcohols. –CH stretch of alkanes was indicated by a peak obtained at around 2940.7691 cm^−1^. A peak for ether functional group was observed at 1041.3721 cm^−1^, describing the presence of C-O-C (Figure 4). Whereas in the case of thiolated hemicellulose, a clear band obtained at around 2667.7691 cm^−1^ indicated the presence of thiol groups (Tlili et al., 2004), hence confirming the successful thiolation of hemicellulose. Moreover, a slightly broad band of –NH2 bending was also seen at 1612.1984 cm^−1^ which was not present in the IR spectra of hemicellulose. This –NH stretch was most likely due to the presence of a primary amine that came from thiourea. Similarly, the (CN) asymmetric stretching mode was also observed at 1471 cm^−1^. The (NH2) asymmetric stretching mode is observed at 3485 cm^−1^. The (NH) stretch mode is observed at 3071 cm^−1^. The C=O stretch of ester exhibited a peak at 1706.6932 cm^−1^ that confirmed the formation of an ester linkage between thiourea and the polymeric backbone. A peak appeared at 1062.5137 cm^−1^ which indicated a C-O-C ether stretch. All these important absorption bands in IR spectra revealed the attachment of a thiol group, hence confirming successful thiolation.

#### Surface Morphology Studies

**FIGURE 5:**
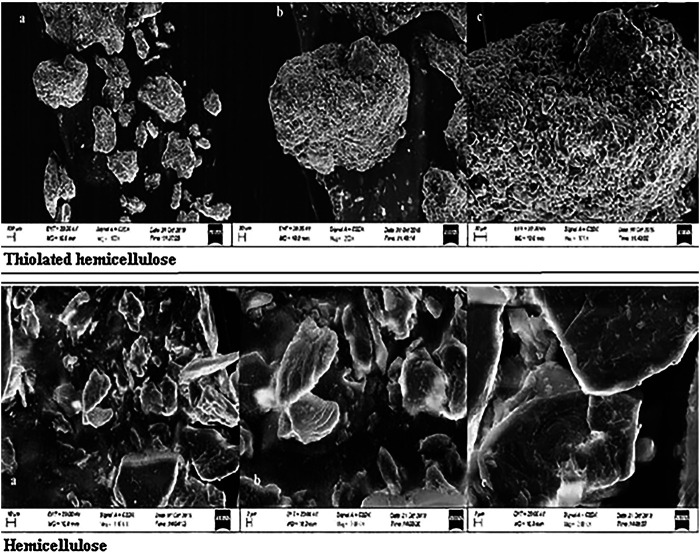
SEM images of Thiolated Hemicellulose and Hemicellulose.

The morphology of the surface was studied using SEM (JSM5910 JEOL, Japan). Surface images for thiolated and non-thiolated polymers were taken at 1000X. These images were used to make a comparative analysis between Hemicellulose and Thiolated hemicellulose. SEM observation revealed that hemicellulose was of irregular shape with different particle sizes whereas thiolated hemicellulose exhibited a crystalline, irregularly shaped material (Figure 5).

#### X-Ray Diffractometry

**FIGURE 6:**
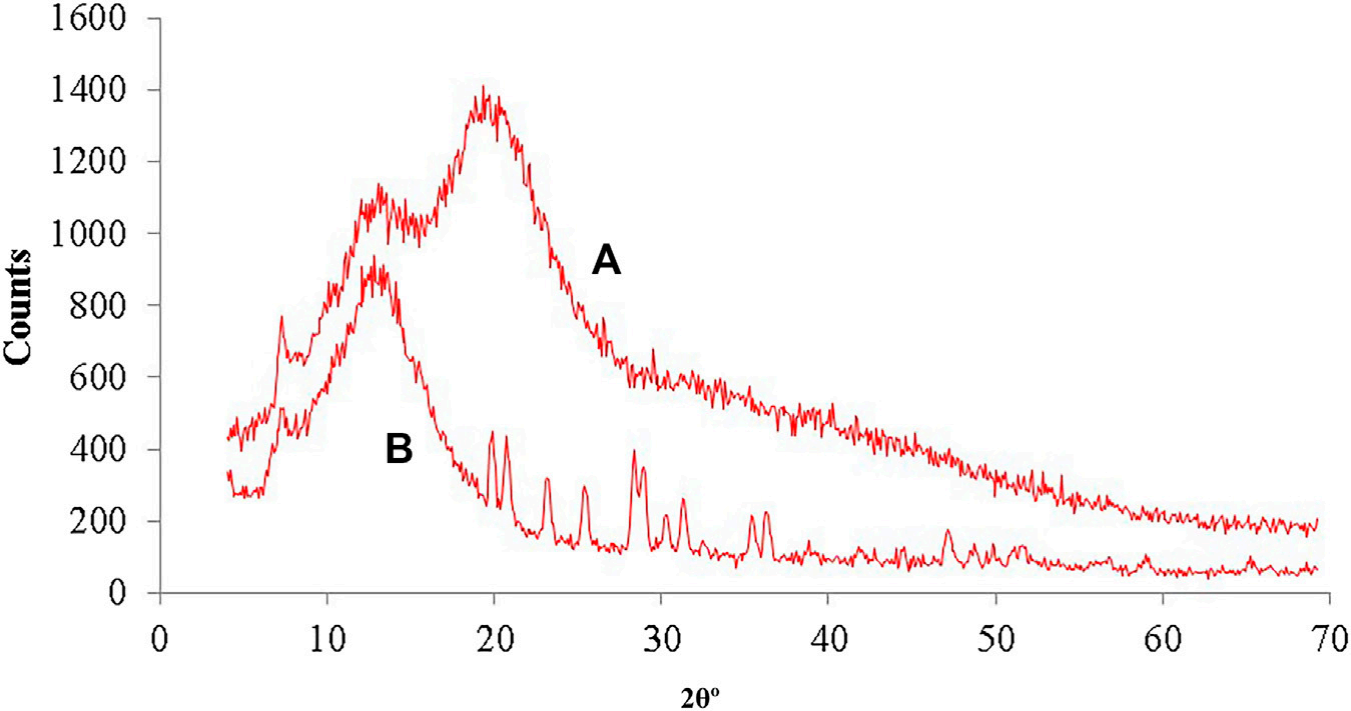
**(A)** XRD diffractogram showing amorphous type nature of Hemicellulose, with various peeks between 20°–50° in XRD of **(B)** Thiolated hemicellulose indicating change in the nature from amorphous to crystalline.

XRD diffraction spectra (Figure 6) of hemicellulose and thiolated hemicellulose demonstrated that no sharp peaks were seen in the diffractgram of hemicellulose exhibiting the amorphous nature of the polymer but three sharp peaks were observed in the diffractgram of thiolated hemicellulose at different positions in the range of 20°, 36°, and 50° (2θ) which clearly indicated that the addition of the thiol group caused a modest proliferation in the crystallinity of hemicellulose. This kind of improvement in the crystallinity of hemicellulose was also reported by Zaman et al. (2018a). The claim of improved crystallinity was also strengthened by the outcomes of XRD, where different sharp peaks were observed in the graph of thiolated hemicellulose. This explains the change in the structure of the polymer, due to the incorporation of thiol moiety.

### Acute Toxicity Studies of Polymer

An acute toxicity study was accomplished to study any kind of toxic effect imparted by Hemicellulose and Thiolated hemicellulose. The results confirmed no toxicity was observed in rats. The signs of any sort of toxicity were absent and all the values obtained from hematology and blood chemistries were within the normal range, advocating no toxicity.

#### Acute Oral Toxicity

During the period of 14 days no mortality was observed in any of the five groups. Treated and untreated rats showed no toxic effect. Physical observation concluded that there was no change in the fur, eyes, behavior pattern, or sleep pattern of the rats.

#### Body Weight

The body weight of all rats of all groups was observed before dosing of the test substances (Hemicellulose and Thiolated hemicellulose) on the third, seventh, and final day. In the first week (up to seventh day), a gradual decrease in the body weight of treated rats was observed, which improved till the 14th day. The body weight observed in Group 1 and Group 2 was not much compared with that of the Control Group (Table 4).

**TABLE 4:** Body weight, Hematology, Blood chemistry, and Relative organ weight of albino rats.

Tests	Parameter/Time	Group 1	Group 2	Group 3
Hypersensitivity	Observed for 24 h	Nil	Nil	Nil
Body Weights	1st day	195	167	164
	3rd day	194	164	161
	7th day	195	162	157
	14th day	196	166	162
Hematology	Hb (g/dl) (10–15 g/dl)	14.1	14.2	13.1
	Total WBCs (x10^3^ μl)	9.7	9.4	9.5
	RBC’s (x10^6^µl)	6.9	7.40	7.25
	Platelets (x10^3^µl)	937	905	789
Blood chemistry Liver profile	AST (U/L)	150	114	114
	ALT (U/L)	42	40	47
	ALP2S (U/L)	129	128.2	124.8
	Bilirubin (mg/dl)	0.03	0.03	0.03
	Total protein (g/dl)	6.0	6.2	5.7
Renal profile	Urea (mg/dl)	27	28	25
	Creatinine (mg/dl)	0.4	0.4	0.3
Relative organ weight (g)	Heart	0.42	0.44	0.410
	Liver	3.70	3.76	3.76
	Kidney	0.401	0.402	0.397

(All values are expressed as mean, n = 3).

#### Hematological and Biochemical Analysis

Hematology and blood chemistry tests were performed in the treated and untreated rats after examining blood samples taken from the rats. The values obtained from the reports were comparable with the values obtained from the control group. Analysis, including complete blood count (CBC), liver profile, and renal profile, have confirmed the safety of the materials as the values obtained were within the normal ranges.

#### Necropsy

All the rats were sacrificed on the 15th day of the study. Three organs, heart, liver, and kidney, were thoroughly examined to find out any kind of abnormality. The relative organ weight was determined for each group at the end of the study, which was later compared with the control group. No significant change was observed. No signs of inflammation and an absence of lesions during the histopathological examination of the heart, liver, and kidney substantiated the safety extent of hemicellulose and thiolated hemicellulose.

#### Relative Organ Weight

Relative organ weight was observed after the dissection of animals. All the weights observed are presented in Table 4 expressed as mean, *n* = 3. In comparison to control all the relative organ weights were in a normal range.

#### Histopathological Evaluation

**FIGURE 7:**
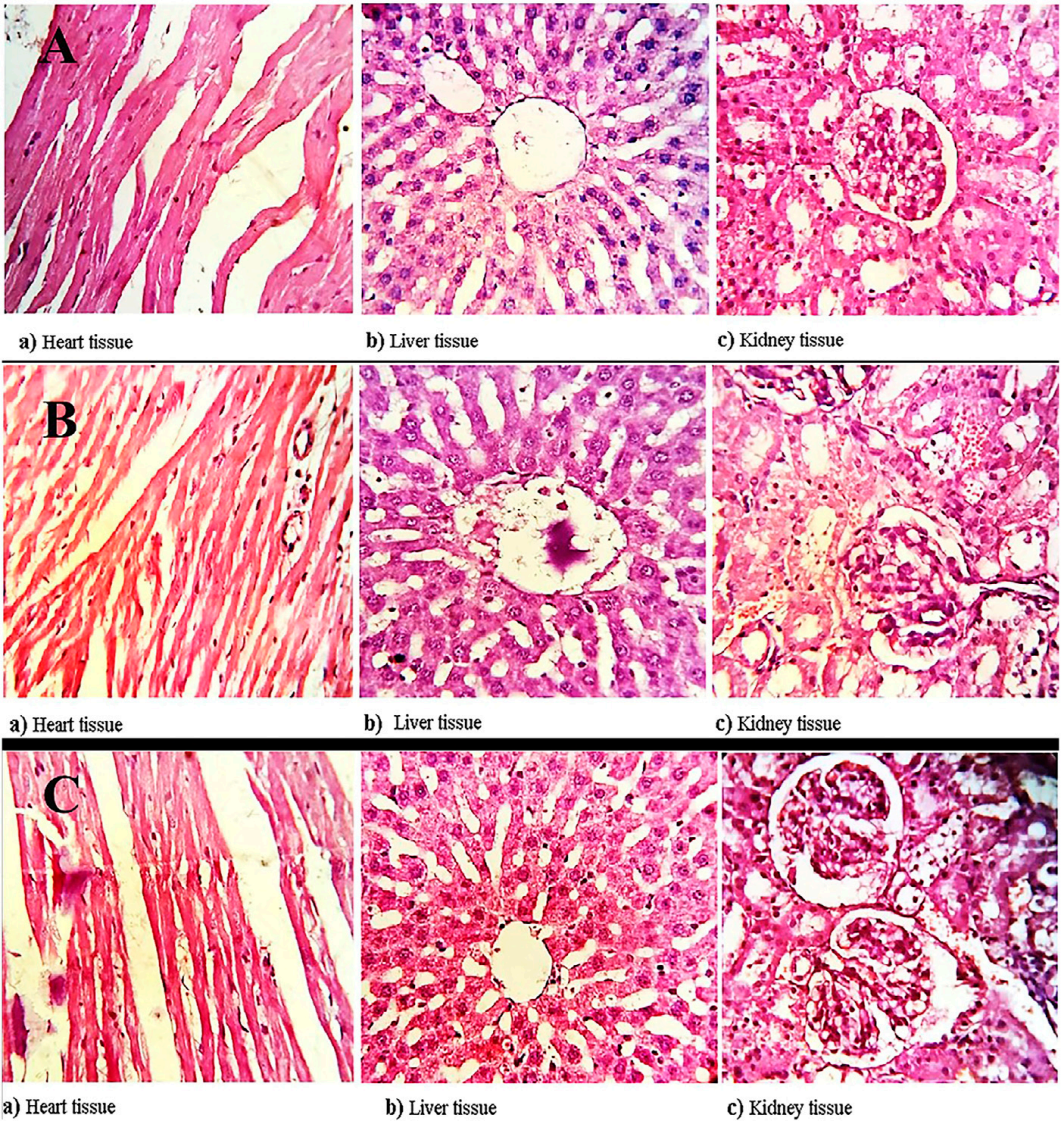
Histopathological evaluation of **(A)** controlled group, **(B)** treated with hemicellulose and **(C)** thiolated hemicellulose, illustrating safety profile of both modified and unmodified polymer, confirming that the polymers are safe to be used in *in-vivo*.

The heart, liver, and kidney of all groups (1, 2, and 3) were washed with saline and preserved in 10% buffered formalin for histopathological examination. The vital tissues embedded in paraffin were stained with hematoxylin and eosin for histopathological evaluation purposes (Saman et al., 2013). All the images were taken on Magnification ( × 400) for control and treated groups (Figure 7). The findings revealed both the modified and un-modified polymers exhibited no adverse effects on the vital organs. All the clinical observations of the treated and control animals were found to be normal (Table 5).

**TABLE 5:** Histopathological evaluation of vital organs including heart, liver, and kidney of the animal.

Group	Tissues	Interpretation
1	Heart	The normal cardiac cells with well defined nucleus observed. The hypertrophy, and the presence of thrombosis as well as infarction was not noticed.
	Liver	The normal tissues, hepatocytes and sinusoidal places were observed. Presence of the microphages in the sinusoidal spaces were observed.
	Kidney	Normal collecting ducts, without any signs of swelling or deposition of lipid droplets were found. Similarly, the intact glomerulus and bowman’s capsule, without having inflammatory invasion was observed.
2	Heart	The normal cardiac cells with well defined nucleus observed. The hypertrophy, and the presence of thrombosis as well as infarction was not noticed.
	Liver	The normal tissues, hepatocytes and sinusoidal places were observed. Presence of the microphages in the sinusoidal spaces were observed.
	Kidney	Normal collecting ducts, without any signs of swelling or deposition of lipid droplets were found. Similarly, the intact glomerulus and bowman’s capsule, without having inflammatory invasion was observed.
3	Heart	The normal cardiac cells with well defined nucleus observed. The hypertrophy, and the presence of thrombosis as well as infarction was not noticed.
	Liver	The normal tissues, hepatocytes and sinusoidal places were observed. Presence of the microphages in the sinusoidal spaces were observed.
	Kidney	Normal collecting ducts, without any signs of swelling or deposition of lipid droplets were found. Similarly, the intact glomerulus and bowman’s capsule, without having inflammatory invasion was observed.

### Ticagrelor Sustained Release Tablets

Direct compression method was used to prepare the modified release tablets of Ticagrelor using hemicellulose and thiolated hemicellulose as formulation Eqs 1, Eqs 2 respectively. The excipients used in the formulations along with thiolated and non-thiolated polymers were polyvinyl pyrolidone, magnesium stearate, aspartame, avicel pH 102, and talc. The compression weight was kept at 250 mg containing 90 mg of Ticagrelor.

#### Post Compression Studies of Ticagrelor Sustained Release Tablets

Tablets prepared from formulations F1 and F2 were subjected to various post compression studies (Table 6) such as diameter/thickness, weight variation, hardness, friability, drug content, disintegration, and *in-vitro* dissolution.

**TABLE 6:** Post Compression studies of Formulations (F1, F2).

Post compression studies	F1	F2
Diameter (mm)	4.6 ± 0.104	4.6 ± 0.109
Thickness (mm)	3.8 ± 0.08	3.8 ± 0.06
Weight variation (%)	Within limit (±7.5%)	Within limit (±7.5%)
Hardness (N)	5.8–6.3 ± 0.012	5.9–6.2 ± 0.10
Disintegration test (min)	425 ± 0.714	480 ± 0.632
Drug content (%)	103.04 ± 2.07	101.09 ± 1.77
Mucoadhesion strength (N)	2.10 ± 0.14	6.23 ± 0.12
Kinetic modeling		
Zero order (R^2^)	0.05	0.1188
First order (R^2^)	0.2575	0.5791
Higuchi model (R^2^)	0.7070	0.7818
Korsmeyer peppas model (R^2^)	0.9883	0.9942
(n)	0.14	0.1

#### Diameter and Thickness

Diameter and Thickness of 10 tablets were measured and the mean diameter and thickness for hemicellulose was found to be 4.6 ± 0.104 and 3.8 ± 0.08. Thiolated hemicellulose was reported to possess a diameter of 4.7 ± 0.109 mm and 3.8 ± 0.06 mm respectively.

#### Weight Variation Test

A sample size of 20 tablets from each formulation F1 and F2 was used for testing weight variation. The weight of tablets varied from 249–254 mg for F1, for that of F2 it varied from 247–251 mg.The average weight was calculated and compared to the individual weight. Low standard deviations were considered. The variation observed among tablets were within range ±7.5% complying with USP specification limits.

#### Hardness

The structural integrity of formulations F1 and F2 were checked with the Monsanto hardness tester. The hardness of F1 formulation was found to be between 5.8–6.3 ± 0.12 kg/cm^3^ 5.9–6.2 ± 0.101 kg/cm^3^ of hardness was reported for F2. F1 and F2 formulations both showed good mechanical integrity.

#### Friability

The mechanical resistance of the tablets was checked by Roche Friabilator, which was allowed to run at 100 RPM for 4 min. The friability was below 1% for both formulations F1 and F2 which indicated good mechanical resistance.

#### Disintegration Test

*In-vitro* disintegration time was noted for six tablets from each formulation (F1, F2). The tablets were allowed to run in DT apparatus under specified conditions, according to the USP disintegration test apparatus. Upon complete disintegration of the tablets, the time was noted and compared. F1 formulation disintegrated within 425 ± 0.714 min, whereas F1 formulation took 480 ± 1.22 min to disintegrate (*n* = 3).

#### Swelling Studies

**FIGURE 8:**
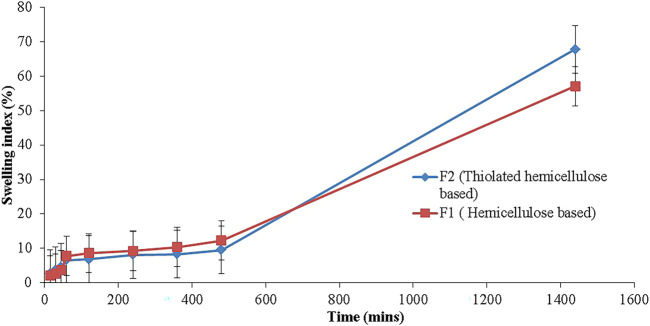
Swelling index of Hemicellulose (F1) and Thiolated Hemicellulose-based tablets (F2).

Swelling studies were conducted to find out the potential of the polymers to absorb and retain the moisture. Swelling is an important parameter to be studied. Hemicellulose-based formulations (F1) showed swelling from the start; after 15 min it showed swelling by 2.1%, which increased with time and progressed to 5.6% till the fourth hour of the study due to the hydration of the polymer. The maximum swelling seen after 24th hour was 57.1%. Whereas, in the case of thiolated hemicellulose-based formulations (F2), swelling after 15 min S.I was about 2.6%, which increased slowly till the fourth hour (8.1%). When the mass was hydrated properly, swelling progressed comparatively in a rapid way and at the end of the 24th hour it was 67.8% (Figure 8).

#### Drug Content (DC%)

The drug content of formulations F1 and F2 were evaluated according to the standard protocols. The results presented in table eight indicate that the percentage drug content was found to be 103.04 ± 2.07% for F1 and 101.09 ± 1.77% for F2 formulation. The results have indicated that the values were well within the acceptable range of USP specifications i.e., 85–115%.

#### *In-vitro* Drug Release Studies

**FIGURE 9:**
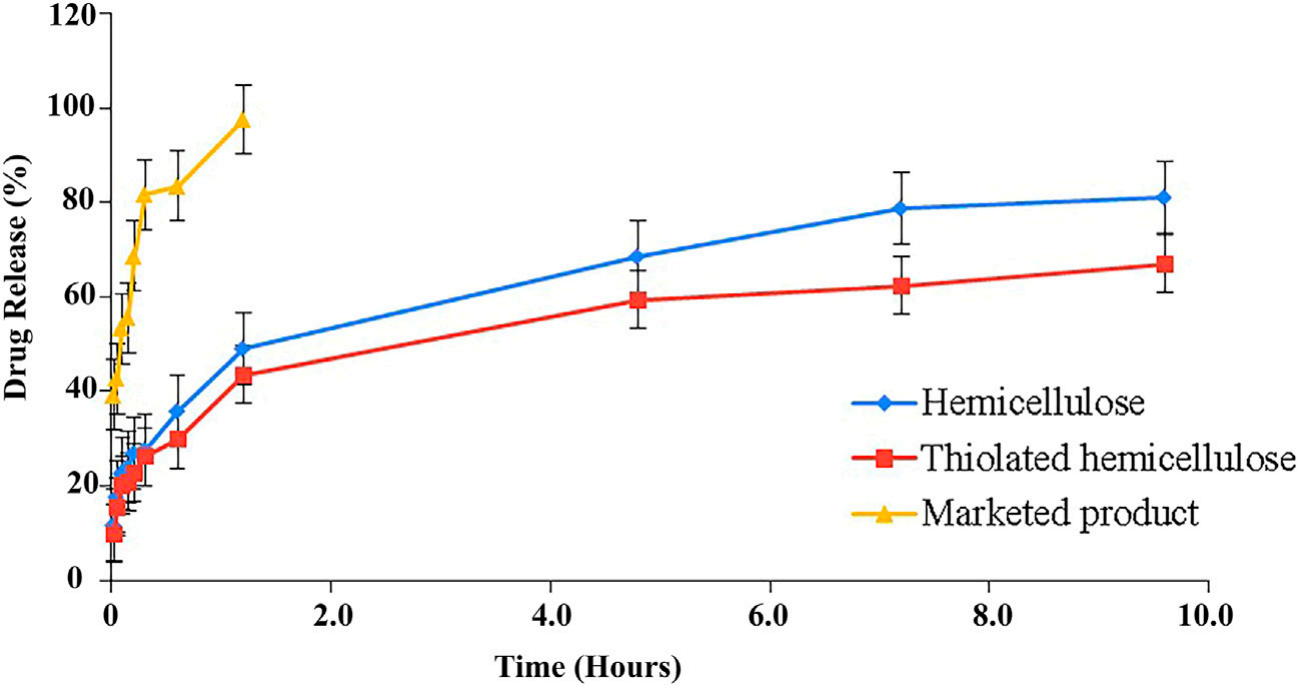
Illustration of dissolution profile of hemicellulose-based (F1) and thiolated hemicellulose-based tablet of Ticagrelor (F2) and their comparison with the % drug release of MP (marketed product).

Dissolution studies have been performed using USP type II dissolution apparatus for 8 h. The study was carried out to determine the release pattern of tablets of both formulations using pH 1.2 acidic buffer. Formulations (F1) and (F2) both showed that there was an initial burst release till the 30th minute of the study, during this 27.62 and 26.12% of the drug was released (Figure 9). There are several factors that could contribute to the initial burst release, including the drug that was attached onto the surface or embedded in the polymer matrix near to the surface. With the passage of time, upon suitable hydration of the matrix, swelling of the polymer started creating a gel layer around the drug particles. Being swellable, polymer thiolated cellulose has the potential to form a thick gel which is basically the cause of drug retardation. Literature has suggested that water swellable polymers block or slow down the release of the drug by creating a barrier. The sustained effect largely depends upon the thickness of the gel layer; as time went on hydration improved, leading to the formation of a more thick and dense layer till the last sample drug was slowly released from the carrier. Greater swelling causes the width of the swollen gelling layer, causing the drug to travel more to reach the surrounding medium. 81.2% drug was released by the formed matrix (F1) and 67.08% from (F2) after 10 h of the study. Thus, the findings advocate hemicellulose and thiolated hemicellulose as potential drug retarding polymers. Moreover, a significantly different release behavior of the drug has been investigated when the release profile of prepared tablets was compared with the marketed product (Figure 9). Around 2 h, all the drug had been excreted from the marketed product, while the prepared tablet provided a good sustained release till the eighth hour of the study.

#### Kinetic Modeling

Release kinetics were evaluated by the application of different kinetic models, namely Zero order, First order, Higuchi, and Korsmeyer Peppas models (Table 6). It was noticed that Korsmeyer Peppas model was a best fit model, suggesting that the release is following diffusion mechanism (F1 and F2). Values of “n” from Korsmeyer model illustrated that the drug release is following a Fick’s law of diffusion *n* = 0.14 (F1), *n* = 0.11 (F2).

#### Mucoadhesion Strength of F1 and F2

The mucoadhesion strength of the formulations (F1, F2) was measured using a Texture analyzer. The results obtained were indicative of an increase in the mucoadhesion strength of hemicellulose upon thiolation (Table 6). Studies were conducted from both formulations i.e. F1 and F2. But it was clear from the results that mucoadhesion improved upon thiolation, as the thiolated formulation (F2) exhibited better mucoadhesion as compared to non-thiolated formulation (F1). Similar mucoadhesion improvement due to thiolation of carboxymethyl dextran was also reported by Kiani et al. (2016).

### Statistical Analysis

Significantly different percentages of cumulative amounts of drug have been released from the three formulations. The findings have disclosed that Thiolated Hemicellulose-based formulation has a better drug retardation effect (*p* < 0.05) as compared to hemicellulose-based formulations and marketed product. F2 has shown significantly different release characteristics from both F1 (*p* = 0.037) and marketed product (*p* = 0.0001).

## Conclusion

The objectives of the studies were to develop biocompatible thiol conjugated hemicellulose, having the potential to act as an effective mucoadhesive pharmaceutical excipient. Successful thiolation was confirmed by the FTIR analysis. The modified polymer has exhibited biocompatibility as well as significantly improved mucoadhesion strength. Furthermore, an improved sustained effect has also been observed during drug release studies. Thiolated polymer, due to its stickier and adhesive nature, may provide the drug with an opportunity to get absorbed from a specific site of the body, and hence, lead to better bioavailability and improved therapeutic effects. In a nutshell, the process opted for thiolation might be considered suitable, and the modification of hemicellulose was found to be useful for the synthesis of a biocompatible pharmaceutical excipient, having potential for use in the delivery of drugs.

## Data Availability

The original contributions presented in the study are included in the article/Supplementary Material, further inquiries can be directed to the corresponding author.
